# Cryoprecipitate as an alternative to platelet transfusion in thrombocytopenia

**DOI:** 10.1002/jha2.358

**Published:** 2021-12-08

**Authors:** Philip Crispin, Sarah Hicks, Lucy A. Coupland, Sidra Ali, Elizabeth E. Gardiner

**Affiliations:** ^1^ ACRF Department of Cancer Biology and Therapeutics The John Curtin School of Medical Research The Australian National University Canberra Australian Capital Territory Australia; ^2^ Department of Clinical Haematology Canberra Hospital Garran Australian Capital Territory Australia

**Keywords:** cryoprecipitate, cryopreserved platelets, thrombocytopenia, thromboelastometry, transfusion

## Abstract

Platelet transfusions are not always available for bleeding in severe thrombocytopenia, as storage outside of major centers is limited by their short shelf‐life. Data are lacking to support alternative available blood products; however, additional fibrinogen has been shown to enhance clot formation in vitro. To test the hypothesis that cryoprecipitate supplementation could improve clot formation in severe thrombocytopenia, eight hematological malignancy patients with platelet counts under 10 × 10^9^/L each had 10 units of apheresis cryoprecipitate transfused prior to planned prophylactic platelet transfusions. The primary endpoint of thromboelastometry amplitude at 20 min increased by a mean of 5.1 mm (*p* < 0.01) following cryoprecipitate transfusion despite persisting thrombocytopenia. Thromboelastometry clotting times reduced by a mean of 7.8 s (*p* < 0.05) and alpha angle increased by a mean of 10.6⁰ (*p* < 0.01). These results are consistent with cryoprecipitate enhancing the strength of the fibrin/platelet meshwork within the forming thrombus. While platelet transfusion remains the standard of care, where platelet supplies are limited, these data provide a rationale for the use of cryoprecipitate to obtain hemostasis in bleeding thrombocytopenic patients.

## 1 INTRODUCTION

Treatment for bleeding in severe thrombocytopenia involves transfusion of room temperature (RT: 20–24°C)‐stored platelets, which have a shelf‐life of 5–7 days due to the risk of bacterial contamination [1]. This complicates platelet stock management and maintaining continuous supplies of platelets is impractical in regional areas with an infrequent need. Nevertheless, bleeding thrombocytopenic patients may still present for care in these centers.

Cryoprecipitate contains high amounts of fibrinogen (15–17 g/L), fibronectin (1500 μg/ml), factor VIII (10.1 IU/mL), von Willebrand factor (VWF 26 IU/mL), and factor XIII (8 IU/mL) [2]. It is the primary source of fibrinogen replacement in many jurisdictions, where fibrinogen concentrate is not widely available or where it is preferred due to lower cost, in the absence of data to support the superiority of fibrinogen concentrate. It can be stored frozen for 12 months, so is not subject to the same fresh supply constraints as platelets and it is stored in regional centers. Fibrinogen concentrate has reduced bleeding in a porcine model of thrombocytopenia [3] and improves thromboelastometry parameters ex vivo [4, 5]. Fibrinogen or cryoprecipitate has not been evaluated for managing hemostasis in severe thrombocytopenia.

Viscoelastic tests, including thromboelastometry, measure the kinetics and strength of clot formation by detecting changes in whole blood elasticity as a clot forms. They have been widely used in the trauma [6, 7, 8] and cardiac surgical [9] settings to predict the risk of bleeding and target goal‐directed therapy. They have also been shown in some studies to predict the risk of bleeding in thrombocytopenia, although data are conflicting [10, 11, 12, 13, 14]. Viscoelastic testing has been shown to be superior to standard clotting tests in some circumstances and because it measures whole blood, may provide a better estimate of overall hemostasis [15, 16].

The current study investigated the potential for cryoprecipitate to improve hemostasis in hypoproliferative thrombocytopenia. We tested the hypothesis that cryoprecipitate transfusion can improve hemostatic parameters in severe thrombocytopenia.

## 2 METHODS

This study was a prospective clinical trial that recruited patients with severe thrombocytopenia prior to prophylactic platelet transfusion. It was prospectively registered at the Australian and New Zealand Clinical Trials Registry (ACTRN12619000322134) and approved by the Australian Capital Territory Health and Australian National University Human Research Ethics Committees and conducted in accordance with the Declaration of Helsinki.

Patients undergoing chemotherapy for hematological malignancies were recruited prior to planned prophylactic platelet transfusion. A history of thrombosis, active sepsis (defined as fever within the previous 48 h), known coagulopathy, and severe bleeding requiring immediate platelet transfusion were exclusions. Patients were transfused with 10 units of apheresis cryoprecipitate prior to the platelet transfusion. The dose of cryoprecipitate was based on approximately 100 mg/kg fibrinogen [4, 5] for a 70 kg recipient and an expected fibrinogen dose of 357 mg/unit [17].

Blood samples were taken before and after each transfusion in EDTA for blood cell enumeration (DXH800, Beckman Coulter, Brea, CA, USA) and in sodium citrate 3.2% (Vacutainer, Becton, Dickinson, Franklin Lakes, NJ, USA) for coagulation, thromboelastometry, flow cytometry, and soluble glycoprotein VI measurement. The coagulation studies performed were prothrombin time (PT, Recombiplastin), activated partial thromboplastin time (APTT, APTT‐SS), and Clauss fibrinogen (QFA Thrombin) all performed on an ACL TOP instrument (Werfen, Barcelona, Spain). APTT clot waveforms were analyzed with data reported using previously recommended nomenclature [18]. Thromboelastometry (ROTEM Delta, Werfen) was performed within 4 h of collection using tissue factor activated (EXTEM), cytochalasin C added and tissue factor activated (FIBTEM), and unactivated (NATEM) tests.

Flow cytometry was performed before and transfusions to detect surface expression of CD42b (GPIbα), GPVI, CD41 (αIIb integrin subunit), CD9, and P‐selectin. Whole blood collected in sodium citrate 3.2% (Vacutainer, Becton, Dickinson) was diluted 1:4 with Tris saline EDTA (or Tris saline CaCl_2_ for tubes with CD62p) and then incubated with AK‐2‐FITC (CD42b, Invitrogen, Waltham, MA, USA), 1G5‐Alexa488 (GPVI, Abcam, Cambridge, UK), CD41‐PE (Abcam), CD‐9 PE (R&D Systems, Minneapolis, MN, USA), and P‐selectin‐PE. Samples were tested on a FACSCalibur (BD Biosciences, San Jose, CA, USA) and data analyzed in Flowing Software v. 2.5.1 (Terho, P, University of Turku, Finland, available at: http://flowingsoftware.btk.fi/).

Soluble GPVI (sGPVI) was measured in citrated platelet poor plasma samples collected and frozen at –80°C at each time point by a sandwich enzyme‐linked immunosorbent assay as previously described [19].

### 2.1 Statistical considerations

The primary endpoint was the change in the EXTEM amplitude at 20 min (A20). With an expected mean pretransfusion level of 27 mm, a standard deviation of 10 mm, and an expected post transfusion increment to a mean of 48 mm [5], eight patients were needed to demonstrate a difference at *p* < 0.05 with 80% power using a two‐sided paired *t* test. Subsequent analyses to compare treatments between the ex vivo or additional ROTEM parameters were considered significant with *p* < 0.05. Paired *t* tests were preferred to compare differences, unless plotted data inspection showed obviously nonparametric data when the Wilcoxin signed rank test was used. Data were analyzed using Prism statistical and graphing software (v6, Graphpad, San Diego, CA, USA).

## 3 RESULTS

### 3.1 Cryoprecipitate transfusion improves thromboelastometry parameters in thrombocytopenia

There were eight (three females) patients recruited, all undergoing chemotherapy for acute myeloid leukemia (6) or non‐Hodgkin lymphoma (2). Bleeding requiring immediate transfusion was an exclusion criterion; however, four had minor bleeding. The median platelet count was 6.5 × 10^9^/L range: 3–9). Four patients had elevated fibrinogen concentrations with a median of 4.4 g/L (1.3–5.2 g/L) and two had a mild prolongation of the PT with a median of 14 s (12–22 s). All patients were anemic with the median hemoglobin concentration of 67.5 g/L (62–98 g/L).

**FIGURE 1:**
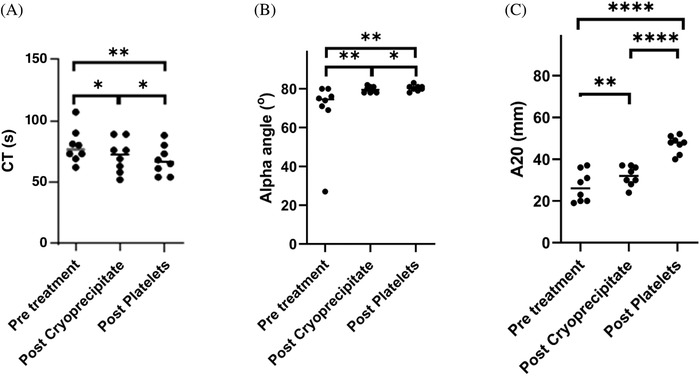
Improvements in thromboelastometry (EXTEM) parameters following treatment with cryoprecipitate and subsequent platelet transfusion (CT: clotting time, A20: amplitude at 20 min). Bar represents the mean value for *n* = 8 individuals; data were analyzed by two‐sided paired *t* test. NS, not significant, * *p* < 0.05, **p < 0.01, ***p < 0.001

**FIGURE 2:**
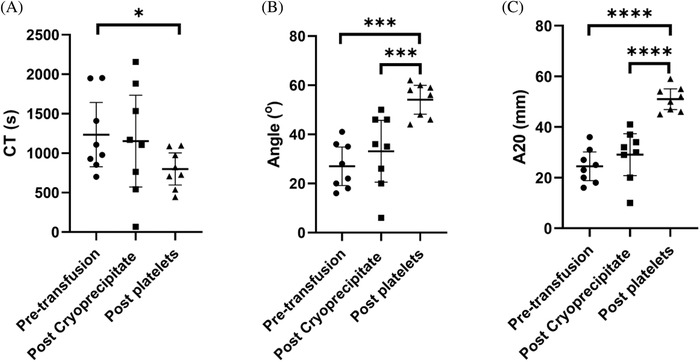
Improvements in native thromboelastometry (NATEM) parameters following cryoprecipitate and subsequent platelet transfusion (CT: clotting time, A20: amplitude at 20 min). Line and error bars represent mean ± SD. Data were analyzed by two‐sided paired *t* test. NS, not significant, * *p* < 0.05, **p < 0.01, ***p < 0.001

Transfusion of cryoprecipitate increased reduced the clotting time (CT) by a mean of 7.8 s (*p* < 0.05) to 71.5 s (Figure 1A). The alpha angle (AA) increased by a mean of 10.6⁰ (*p* < 0.01) to 79.6⁰ (Figure 1B). The EXTEM A20 increased by a mean of 5.1 mm (*p* < 0.01, Figure 1C) to 32 mm. There was a mean increment in fibrinogen of 1.59 g/L and the FIBTEM A20 increased by 7.5 mm (*p* < 0.001) to be above the reference range in all patients. The FIBTEM CT (8.4 s, *p* < 0.05) and AA (4⁰, *p* < 0.01) also improved following cryoprecipitate infusion. There was no significant change in any NATEM parameters with cryoprecipitate transfusion (Figure 2).

Platelet transfusion occurred as soon as possible after cryoprecipitate transfusion and subsequent blood sampling, and led to a mean platelet increment of 26.4 × 10^9^/L to a mean of 33.4 × 10^9^/L. Compared to the preplatelet transfusion samples, there was a significant increase in A20 (mean increase 15 mm, *p* < 10^–4^ Figure 1C) and amplitudes at all other time points. Platelet transfusion further improved the CT by 3.6 s (*p* < 0.05, Figure 1A) and the AA by 0.75⁰ (*p* < 0.05, Figure 1B).

The FIBTEM showed no further increase after platelet transfusion for any parameters. NATEM showed improvements with platelet transfusion not observed with cryoprecipitate with a mean improvement in A20 of 21.9 mm (*p* < 0.001, Figure 2C) and AA of 21⁰ (*p* < 0.001, Figure 2B), but the change in CT between postcryoprecipitate and postplatelet samples was not significant (Figure 2A). There was no evidence of clot lysis in any sample.

### 3.2 Platelet surface markers and APTT clot curves following transfusion

Surface levels of platelet receptors can reflect platelet quality, functionality, and level of activation. Platelet flow cytometry data were available on seven of the eight transfused patients and are shown in Table 1. There was no significant change in platelet surface protein levels following cryoprecipitate transfusion for any of the measured proteins. While there was an increase in the proportion of GPIbα and GPVI‐positive events within the low forward scatter/low‐side scatter gate following platelet transfusion, this was not seen following correction for the increased CD41a‐positive events. P‐selectin‐positive platelets increased following platelet transfusion from 28% to 56% (*p* < 0.01). Although there was no increase in the proportion of GPVI‐positive platelets, there was a significant increase in the geometric mean of GPVI expression on platelets following platelet transfusion (348–1018, *p* < 0.02, Table 1). As the platelet increment was greater than the initial platelet count, postplatelet transfusion results are likely to reflect the platelet surface protein levels of transfused stored platelets.

**TABLE 1:** Changes in platelet antigen with transfusion

	Pretransfusion		Postcryoprecipitate		Postplatelet transfusion	
Antigen	Proportion positive (mean %)	Geometric mean	Proportion positive (%)	Geometric mean	Proportion positive (%)	Geometric mean
αIIb integrin	59.6	2068	51.7	2007	92.8^a^	2089
CD9	44.2	80.3	33.4	82.6	66.7^b^	43.5
GPVI	57.5	392	45.5	348	91.4^a^	429^d^
GPIbα	46.7	1233	45.1	933	92.6^a^	1018
P‐selectin	16.4	127	15.0	93	51.0^c^	82

^a^ Significantly different to both pretransfusion and postcryoprecipitate samples, but not significant when corrected for CD41‐positive events.

^b^ Significantly different from postcryoprecipitate sample, but not significant when corrected for CD41‐positive events.

^c^ Significantly different to both pre and post transfusions.

^d^ Significantly different from postcryoprecipitate sample.

**FIGURE 3:**
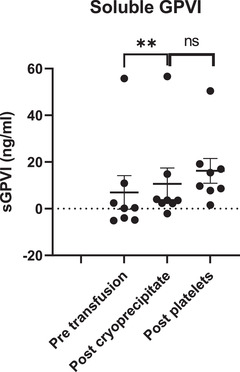
Changes in plasma soluble GPVI with transfusion as measured by ELISA for *n* = 7–8 individuals. Line and error bars represent mean ± SD. Data were analyzed by two‐sided paired *t* test. NS, not significant, ** *p* < 0.01

Levels of sGPVI (the soluble ectodomain of GPVI released from activated platelets by metalloproteolysis) in plasma pretransfusion fell within normal ranges observed in healthy donors [20], and showed a mean increase of 3.7 ng/ml from pretransfusion levels following cryoprecipitate transfusion (*p* = 0.002) and a smaller increment following subsequent platelet transfusion, which did not reach statistical significance (*p* = 0.06, Figure 3). sGPVI was subsequently measured in four apheresis cryoprecipitate units, showing a mean of 32.1 ng/ml. This would increase sGPVI by 5.7 ng/ml with a 10 unit (600 ml) transfusion into a 4000 ml plasma volume, suggesting that sGPVI changes were related to sGPVI in transfused cryoprecipitate rather than metalloproteolysis from endogenous platelets [21].

**FIGURE 4:**
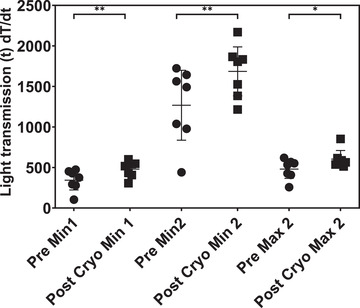
APTT clot curve parameters before and after cryoprecipitate (cryo) transfusion. Min 1 is maximal rate of clot formation. Min 2 is maximal rate of acceleration of clot formation. Max 2 is maximal rate of clot deceleration. Line and error bars represent mean ± SD. Data were analyzed by two‐sided paired *t* test; ** *p* < 0.01, * *p* < 0.05

The PT and APTT did not change significantly following cryoprecipitate transfusion; however, the analysis of APTT clot waveforms did show significant changes (Figure 4). The maximum clot formation velocity (Min 1) showed a significant increase (*p* = 0.001) as did the maximum clot acceleration (Min 2, *p* < 0.005) and the maximum deceleration at the end of clot formation (Max 2, *p* < 0.05).

## 4 DISCUSSION

The perishable nature of RT‐stored platelets limits their availability for urgent transfusion, especially in rural or remote areas. This study explored the potential for cryoprecipitate, which has a longer shelf‐life, to replace RT‐stored platelets in severe thrombocytopenia. It showed that thromboelastometry parameters improved without correcting thrombocytopenia and provides a rationale for cryoprecipitate in bleeding thrombocytopenic patients when fresh platelets are not available.

Prior studies have compared the addition of fibrinogen concentrate ex vivo with in vivo platelet transfusion and both showed similar efficacy on thromboelastometry amplitudes [4, 5]. Transfusion into severely thrombocytopenic patients led to improvements in the thromboelastometry A20, as well as CT and AA, and confirmed their potential role in achieving hemostasis. This indicates that cryoprecipitate enhanced both the rate and extent of clot formation. The improvement in AA and APTT clot formation rate suggests enhanced thrombin activity following the addition of cryoprecipitate and the enhanced EXTEM amplitudes suggest greater total clot formation.

The formation of stable clot requires the formation of a fibrin meshwork, within which platelets are intertwined contributing to clot retraction. This is important in promoting local fibrin formation by slowing flow of solutes away from the site of injury [22]. Other clotting factors, platelet surface phosphatidylserine, platelet receptors that actively bind VWF, fibrinogen, and fibrin (including GPIbα, GPVI, and αIIbβ3), and the rate of clot formation all contribute to attain the final clot strength. However, fibrin with platelets constitutes the final clot and this study shows that the improvement in clot strength may be achieved in severe thrombocytopenia by increasing the fibrinogen concentration. Importantly, this effect was seen even when the baseline fibrinogen concentration was elevated, as may be expected in patients with hematological malignancy.

The study was not designed to compare platelet with cryoprecipitate transfusion, since the transfusion of platelets was always subsequent to cryoprecipitate. However, small further improvements in AA and CT were noted and a substantial increase in A20 occurred following RT‐stored platelet transfusion, as expected in severe thrombocytopenia. Where platelet concentrates are available, they remain the treatment of choice for treating bleeding in severe thrombocytopenia. The enhancement of coagulation clot formation rate and strength despite thrombocytopenia may explain the benefit of goal‐directed fibrinogen replacement in the critical bleeding setting [8].

The main limitation of this study is the use of surrogate endpoints. Correction of thromboelastometry parameters has been used to inform the management of bleeding in other settings [23]. While thromboelastometry uses whole blood, it does not measure rheology‐induced changes or the contribution of endothelial cells and there remains uncertainty, despite the primacy of correcting amplitudes in clinical bleeding protocols, of what parameters best correlate with bleeding. Variable association between thromboelastometry amplitudes and bleeding in thrombocytopenic patients has been observed [10, 11, 12, 13, 14]. This limits the confidence with which clinical recommendations can be made, however, provides a rationale for therapy when platelet concentrates are not available, especially noting the difficulty of clinical trials in this setting.

In conclusion, these results indicate that cryoprecipitate transfusions may be of value to improve hemostasis in severe thrombocytopenia. While it will be important to determine prospectively whether cryoprecipitate stops bleeding, these studies offer a rationale for its use in bleeding thrombocytopenic patients when platelets are not immediately available for transfusion.

## CONFLICTS OF INTEREST

PC has received support from CSL Behring for research consumables and travel. There are no other conflicts of interest to declare.
